# Pre and postoperative value of contrast computerized tomography scan for patients with abdominal aorta aneurysm treated with endovascular repair study

**DOI:** 10.1186/1749-8090-10-S1-A231

**Published:** 2015-12-16

**Authors:** Serkan Yazman, İsmail Yürekli, Levent Yılık, Ufuk Yetkin, Köksal Dönmez, Hasan İner, Tevfik Güneş, Barçın Özcem, Ali Gürbüz

**Affiliations:** 1 Department of Cardiovascular Surgery, Katip Celebi University Izmir Ataturk Training and Research Hospital, Izmir, Turkey

## Background/Introduction

One of the most important advantages of endovascular treatment is decreased systemic complication rates, especially complications in perioperative period, against conventional surgery.

## Aims/Objectives

We included 203 abdominal aortic aneurysm patients treated with endovascular aortic repair (EVAR) between dates 2006 and 2013.

## Method

All of the patients had high risk for conventional surgery. Mean age was 69.17 ± 8.83 (between 38-89). Sixteen of these patients were female (7,9%) and 187 of them were male (92,1%). Twelve patients underwent emergency surgery for ruptured abdominal aortic aneurysm. Any additional pathology that may interfere conventional surgery and increase the operative risk was investigated and effected patient selection.

## Results

Treatments of all patients were planned by using contrast computerized tomography. Inclusion criteria were; asymptomatic patients with abdominal aorta diameter on CT equal to or larger than 5,5 cm, symptomatic patients with abdominal aorta diameter on CT smaller larger than 5,5 cm, patients with saccular aneurysm and ruptured abdominal aorta aneurysm.

## Discussion/Conclusion

Ultrasonography is safely used in first diagnosis, follow-up and postoperative controls. Preoperative evaluation, imaging of abnormalities, identifying ruptures and localization of aneurysm may be performed on contrast CT. Due to different complication after EVAR, yearly radiologic follow-ups by computerized tomography, ultrasonography and direct x-rays are essential.

## Consent

Written informed consent was obtained from the patient for publication of this Case report and any accompanying images. A copy of the written consent is available for review by the Editor-in-Chief of this journal.

